# Optical bone densitometry robust to variation of soft tissue using machine learning techniques: validation by Monte Carlo simulation

**DOI:** 10.1117/1.JBO.27.5.056004

**Published:** 2022-05-18

**Authors:** Kaname Miura, Anak Khantachawana, Shigeo M. Tanaka

**Affiliations:** a Kanazawa University, Graduate School of Natural Science and Technology, Division of Mechanical Science and Engineering, Kanazawa, Japan; b King Mongkut’s University of Technology Thonburi, Faculty of Engineering, Biological Engineering Program, Bangkok, Thailand; c King Mongkut’s University of Technology Thonburi, Department of Mechanical Engineering, Faculty of Engineering, Bangkok, Thailand; d Kanazawa University, Institute of Science and Engineering, Faculty of Frontier Engineering, Kanazawa, Japan

**Keywords:** optics, osteoporosis, bone densitometry, Monte Carlo simulation, machine learning, reaction-diffusion model

## Abstract

**Significance:**

To achieve early detection of osteoporosis, a simple bone densitometry method using optics was proposed. However, individual differences in soft tissue structure and optical properties can cause errors in quantitative bone densitometry. Therefore, developing optical bone densitometry that is robust to soft tissue variations is important for the early detection of osteoporosis.

**Aim:**

The purpose of this study was to develop an optical bone densitometer that is insensitive to soft tissue, using Monte Carlo simulation and machine learning techniques, and to verify its feasibility.

**Approach:**

We propose a method to measure spatially resolved diffuse light from three directions of the biological tissue model and used machine learning techniques to predict bone density from these data. The three directions are backward, forward, and lateral to the direction of ballistic light irradiation. The method was validated using Monte Carlo simulations using synthetic biological tissue models with 1211 different random structural and optical properties.

**Results:**

The results were computed after a 10-fold cross-validation. From the simulated optical data, the machine learning model predicted bone density with a coefficient of determination of 0.760.

**Conclusions:**

The optical bone densitometry method proposed in this study was found to be robust against individual differences in soft tissue.

## 1 Introduction

Bone strength is determined by two factors: bone mineral density (BMD), which is a measure of bone mass, and bone quality, which is a measure of bone structure and microfractures.^1^ Osteoporosis is a disease in which the bone strength is reduced, thereby increasing the risk of fracture.^1^ Bone mass increases during growth, peaks in the twenties, and declines with age from around the forties.^2^ However, failure to achieve sufficient peak bone mass increases the risk of future osteoporosis. Fractures due to osteoporosis reduce the quality of life^3^^,^^4^ and, in the long term, significantly increase the risk of mortality, regardless of the presence or absence of fractures.^5^^,^^6^ Osteoporosis is called the “silent disease” because there are often no subjective symptoms even when bone mass is reduced.^7^ In addition, two-thirds of hip fracture patients will never regain their previous activity level.^1^ Accordingly, early detection of a decrease in BMD might be an effective tool for early intervention.

The gold standard for BMD measurements is double energy x-ray absorptiometry (DXA), which quantifies BMD as areal BMD (aBMD, $g/{\mathrm{cm}}^{2}$).^1^^,^^8^^,^^9^ Although DXA has excellent fracture prediction capabilities,^9^^–^^11^ its application to the early detection of low BMD is limited by its large size and the risk of exposure to ionizing radiation. Quantitative ultrasound (QUS) is the only method for measuring bone without ionizing radiation and may predict fracture risk independently of DXA.^1^^,^^12^ However, QUS scores are distinct from measurements based on BMD, and diagnostic criteria have not been defined. Motivated by the need to measure BMD in a simple and safe manner for the early detection of osteoporosis, optical bone densitometry has been studied.^13^^,^^14^

Bone densitometry using near-infrared light could be a new screening method for osteoporosis. Near-infrared light has excellent biological penetration properties,^15^ and there is a strong relationship between BMD and light scattering phenomena.^16^^,^^17^ The bone matrix is composed of calcium-containing hydroxyapatite crystals deposited on a collagen fiber matrix. The principle of BMD measurements using x-rays, including DXA, is based on the linear relationship between the concentration of the crystal and the absorption of x-rays. The bone also intensely scatters optical photons because of these crystals. An early *in vitro* study by Takeuchi et al.^18^ showed a strong correlation between transmitted light intensity and aBMD. In addition, Ugryumova et al.^16^ showed that the scattering coefficient correlates with compact bone BMD on visible and near-infrared wavelengths.

Despite the strong relationship between BMD and light scattering, the bone matrix is not the only substance that scatters light in biological tissues. Most bones are at least covered by soft tissues, such as the dermis and subcutaneous tissue. In the visible and near-infrared wavelength range, quantitative measurement of BMD is difficult because of variations in soft tissue structure and optical properties caused by individual differences. For example, Pifferi et al. used time-resolved transmittance spectroscopy (TSR) to measure the calcaneus; however, they demonstrated that large measurement errors can occur because of differences among subjects and the complexity of the soft tissue.^19^ In addition, Chung et al. reported a correlation between near-infrared light transmittance and aBMD in the ultradistal radius, but they were concerned about the bias from soft tissue.^14^ We previously reported that the slope of the intensity distribution formed by diffuse reflected light correlates with BMD but showed nonlinear variation with skin thickness and BMD.^13^

We propose the following approach to solve the problem of variation in optical measurements resulting from individual differences in soft tissue. First, spatially resolved steady-state diffuse light was measured in three directions in the medium. Here, the three directions are backward, forward, and lateral to the direction of ballistic light irradiation. The method is based on the idea that there is a functional relationship between the measurement direction and light scattering by the bone. In addition, the penetration depth of the light reflected in the backward direction corresponds to approximately half of the distance between the light source and the detector,^20^ and the light observed in the forward and lateral directions reflects all information on the light path to get there. Specifically, diffuse light spatially resolved in different directions provides mixed information about the structure and optical properties of all tissues through which the light passes, with different degrees of influence depending on the location being measured. Second, the relationship between diffuse light and BMD acquired at different positions was generalized using machine learning (ML) techniques. ML is a method of analyzing data in which a computer automatically learns and discovers the rules and patterns behind the data.^21^ For an ML model to have sufficient generalization performance, it needs data from a population with sufficient variance to represent individual differences in soft tissue and BMD.

Biological tissue models with random structures and optical properties were generated, and the light transport was simulated using the Monte Carlo method. The biological tissue model consisted of the dermis, subcutaneous tissue, and bone tissue. The structural and optical properties of the soft tissue were randomly determined to represent a population with sufficient variance. In the bone tissue, the three-dimensional (3D) trabecular pattern was generated using Alan Turing’s reaction-diffusion model^22^ because it is difficult to assign the trabecular bone a single optical property representative of a BMD value. The reaction-diffusion model is a widely used mathematical theory for describing the pattern formation process in biology, which is observed in bone during the early stages of calcification and development.^23^^,^^24^ Because trabecular bone has a 3D nonlinear structure, the simulation was performed using a voxel-based Monte Carlo (VMC) model, which is a 3D extension of the Monte Carlo model for steady-state light transport in multilayered tissues (MCML) by Wang et al.^25^^,^^26^

The purpose of this study is to develop and validate an ML prediction model with diffuse light as training data using a Monte Carlo simulation. The Monte Carlo model simulated spatially resolved steady-state diffuse light data acquired in three directions of the biological tissue model assuming an ultradistal radius. If this method is sufficiently accurate, it can be used for simple and safe bone densitometry, thus contributing to the early detection of osteoporosis.

## 2 Materials and Methods

### 2.1 Generation of Biological Tissue Model

**Fig. 1:**
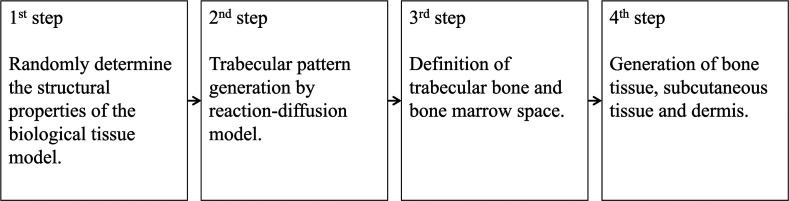
Procedure for generating tissue models.

The procedure for generating the biological tissue model is shown in Fig. 1. The biological tissue model consisted of the bone tissue, subcutaneous tissue, and dermis. The bone tissue was composed of the cortical bone, trabecular bone, and bone marrow. In this section, we describe the four steps for generating the biological tissue model.

In the first step, the structural properties of the biological tissue model were determined randomly. The structural properties of the biological tissue models are listed in Table 1. The thicknesses of the dermis and the subcutaneous tissue were determined using random numbers of uniform probability with a range of 1 to 2 mm^27^ and 1 to 6 mm,^28^ respectively. The structural properties of the bone tissue were based on measurements of the ultradistal radius of women by Boutroy et al.^29^ The cortical bone thickness (C.Th) and trabecular bone volume (BV) ratio [BV/tissue volume (TV)] vary at different stages of osteoporosis (osteoporosis, osteopenia, and healthy subjects). Therefore, the C.Th and BV/TV were determined using random numbers, assuming a normal distribution with different mean and variance values for each stage of osteoporosis. The distributions of C.Th and BV/TV were correlated with a Pearson correlation coefficient r of 0.54. The BMD of bone matrix (mBMD) assumed $1.2\;g/{\mathrm{cm}}^{3}$ of fully calcified bone.

**Table 1:** Structural properties of biological tissue models.

	Thickness in z- and x-axis direction (mm)	BV/TV	mBMD ($g/{\mathrm{cm}}^{3}$)	Ref.
Dermis	1.0 to 2.0	—	—	Kozarova et al.^27^
Subcutaneous	1.0 to 6.0	—	—	Hassager et al.^28^
Cortical bone	Osteoporosis 0.487 ± 0.138, osteopenia 0.571 ± 0.173, normal 0.804 ± 0.149.	—	1.2	Boutroy et al.^29^
Trabecular bone	17.15 minus the cortical bone thickness	Osteoporosis 8.5 ± 2.2, osteopenia 10.3 ± 3.0, normal 13.4 ± 2.8	1.2	Boutroy et al.^29^

The values showing the range (number–number) have a uniform distribution of probability, and the mean ± standard deviation has a normal distribution. The three states of cortical bone thickness and BV/TV correspond to their respective values.

In the second step, the trabecular pattern was generated using Alan Turing’s^22^ reaction-diffusion model. The modeling was based on a report by Miura and Maini.^30^ The governing equation of the diffusion-reaction model is

$$\left\{ \begin{array}{c} \frac{\partial u}{\partial t}=f(u,v)+du\Delta u\\ \frac{\partial v}{\partial t}=g(u,v)+dv\Delta v \end{array}.\right.$$ (1)

This equation is called the activator-inhibitor system and is represented by the nonlinear reaction functions f(u,v) and g(u,v), as well as diffusion terms, where u is the concentration of the activator and v is the inhibitor. The nonlinear terms f and g are

$$f=0.6u-v-u^{3},\;g=1.5u-2v.$$ (2)

The diffusion term Δ is the Laplace operator, and du and dv are the diffusion coefficients. In this study, du and dv were set to 0.0002 and 0.01, respectively. The governing equation [Eq. (1)] was solved by an implicit scheme^31^ based on the Fourier transform on a 0.025 grid in a 720×720×720 3D grid space, assuming periodic boundary conditions. The calculation was repeated 100 times with dt=1, and the solution was confirmed to converge. The initial states of u and v were assumed to be uniformly distributed random numbers within a range of ±0.5.

**Fig. 2:**
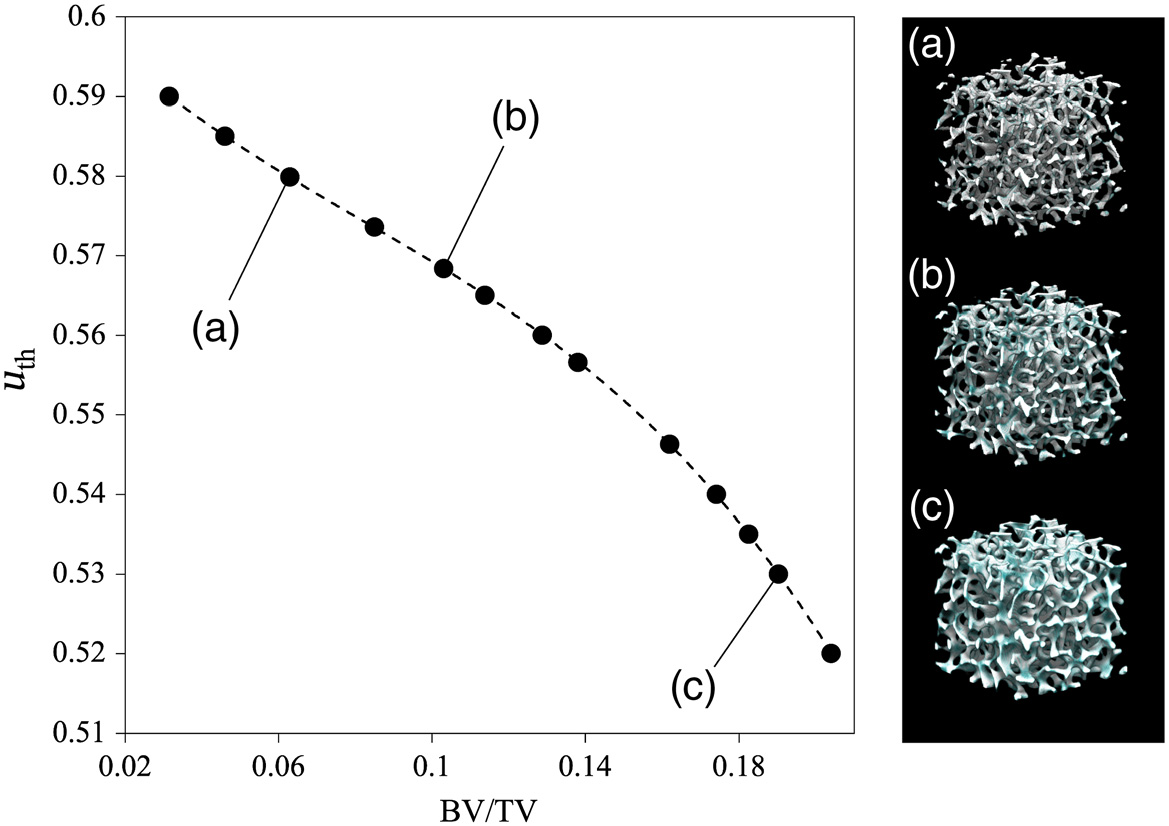
Relationship between BV/TV and $u_{\mathrm{th}}$, where $u_{\mathrm{th}}$ is the threshold of the activation factor u.

In the first step, the border between the trabecular bone and bone marrow space was defined by the threshold $u_{th}$. First, a $u_{th}$ was applied to ten trabecular patterns, which were determined by randomly generating u with 10 different initial conditions. Accordingly, 10 trabecular bones were created with a similar BV/TV, in which $u\geq u_{th}$ was defined as the trabecular bone and $u<u_{th}$ as marrow space. By repeating this process with 13 $u_{th}s$, as shown in Fig. 2, the relationship between $u_{th}$ and BV/TV was obtained and is expressed as follows:

$$u_{th}=-7.662{\left(BV/TV\right)}^{3}+1.645{\left(BV/TV\right)}^{2}-0.452BV/TV+0.604.$$ (3)

**Fig. 3:**
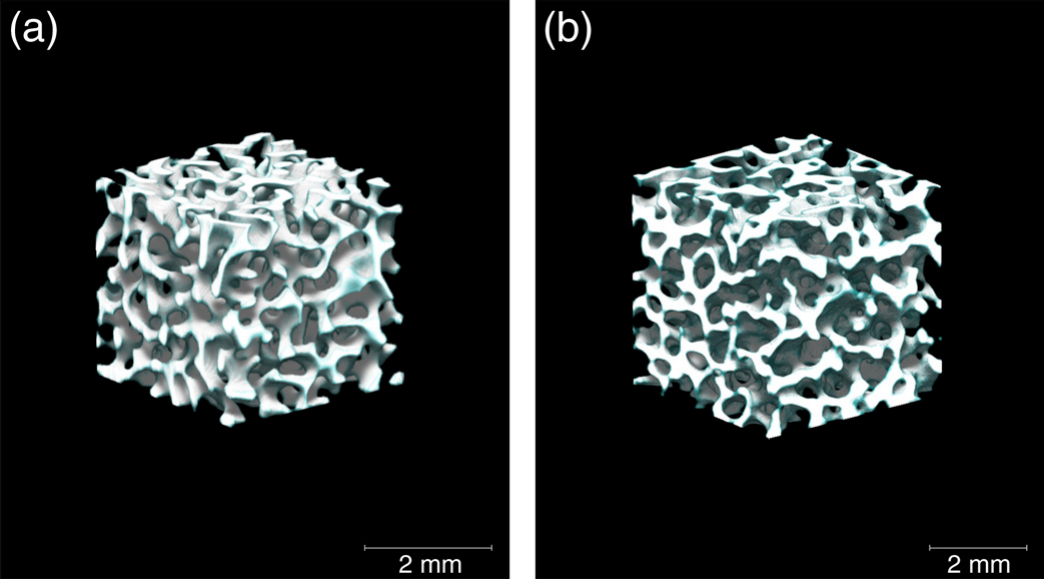
Comparison of appearance between (a) trabecular bone generated by the reaction-diffusion model and (b) a μCT image of a bovine trabecular bone taken from a femoral neck.

Equation (3) was fitted to a polynomial function using the least-squares method. Next, the voxel size u was defined. Because u is composed of voxels that do not have a size, we set the voxel size to 24.5  μm. This voxel size was similar to the resolution of the μCT image. The generated trabecular bone appeared to replicate the trabecular pattern observed in the real bone, as shown in Fig. 3. In addition, the generated trabecular bone was comparable to the ultradistal radius with respect to the trabecular number (Tb.N, ${\mathrm{mm}}^{-1}$),^29^ fractal dimension,^32^^,^^33^ and structure model index (SMI),^34^^,^^35^ as shown in Table 2. These values quantitatively evaluate the trabecular bone shape. Because of the anisotropy of the trabecular pattern, the trabecular space (Tb.Sp) and variance (Tb.Sp SD), as well as the trabecular thickness (Tb.Th), were smaller than those of the ultradistal radius. In this study, this model was selected as the trabecular bone model because it can represent BMD changes in BV/TV, and the trabecular pattern resembles the shape of the real bone. Bone histomorphometry measurements were derived using TRI/3D-BON-FCS (Ratoc System Engineering Co. Ltd., Japan).

**Table 2:** Comparison of measurements of bone histomorphometry between the trabecular bone generated by the reaction–diffusion model and that of the human ultradistal radius.

	Generated trabecular bone			Trabecular bone of the ultradistal radius			
	Normal	Osteopenia	Osteoporosis	Normal	Osteopenia	Osteoporosis	Ref.
BV/TV (%)	13.4	10.3	8.5	13.4 ± 2.8	10.3 ± 3.0	8.5 ± 2.2	Boutroy et al.^29^
Tb.N (${\mathrm{mm}}^{-1}$)	1.74	1.51	1.32	1.71 ± 0.22	1.44 ± 0.29	1.32 ± 0.21	
Tb.Th (μm)	56	52	49	78 ± 11	71 ± 11	63 ± 11	
Tb.Sp (μm)	204	211	219	517 ± 88	656 ± 187	714 ± 140	
Tb.Sp SD (μm)	40.6	42.5	44.3	212 ± 58	342 ± 201	340 ± 89	
Fractal dimension	2.13	2.03	1.93	2.33 ± 0.04		2.24 ± 0.03	Bayarri et al.^33^
SMI	2.04	2.27	2.44	2.26 ± 0.38			Zhou et al.^35^
Degree of anisotropy	1.01	1.02	1.03	1.45 ± 0.09			

**Fig. 4:**
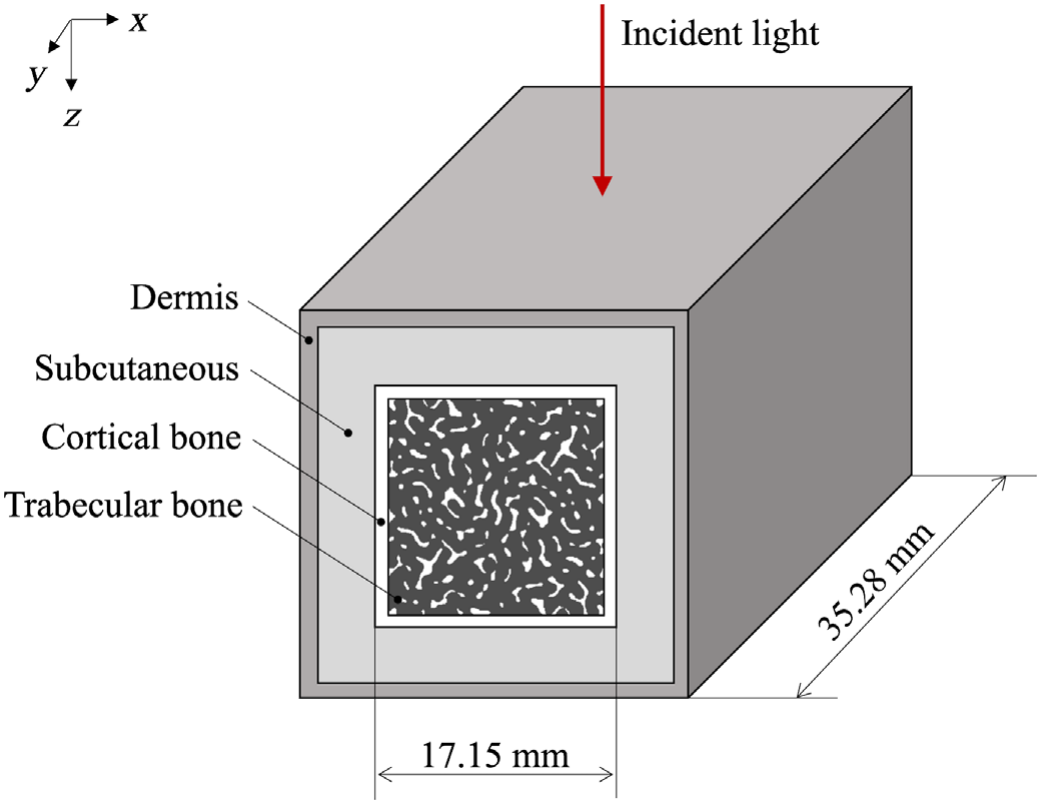
Appearance of the synthetic biological tissue model assuming the ultradistal radius.

In the fourth step, the bone tissue, subcutaneous tissue, and dermis were defined, and a biological tissue model was generated, as shown in Fig. 4. The biological tissue model was assumed to be the ultradistal radius, and the bone axial direction was set as the y axis. First, the size of bone tissue was determined. To ensure sufficient size in the bone axial direction, the generated trabecular bone was copied and joined in the y axis direction. From the cross-sectional area of the ultradistal radius,^29^ a square with a size of 17.15 mm per side was defined as the outer bone surface (OBS) in the x-z plane (Fig. 4). Thus, the size of the bone tissue was 17.15 mm on the x-z axis and 35.28 mm on the y-axis. This size is comparable to the area of the trabecular bone in the distal radius.^29^ Next, a cortical bone with a size of C.Th was defined from the OBS toward the center of the trabecular bone, and the bone tissue was generated. The aBMD of the bone tissue was defined using BV/TV, the size of the OBS in the x-axis direction ($l_{\mathrm{obs}}$, cm), and mBMD as follows:

$$\mathrm{aBMD}=\mathrm{mBMD}[1-(1-BV/TV){\left(1-\frac{2C.Th}{l_{\mathrm{obs}}}\right)}^{2}].$$ (4)

Finally, the subcutaneous tissue and dermis were generated in order from the OBS to the outward direction of the bone tissue, as shown in Fig. 4. All of the above processes were coded in Python 3.8.

### 2.2 Monte Carlo Simulation

Light transport in the synthetic biological tissue model was simulated using the Monte Carlo method. To deal with a tissue model containing trabecular bone with a 3D nonlinear pattern (Fig. 4), a VMC was built by extending MCML^25^^,^^26^ to three dimensions. To represent individual differences, the optical properties of soft tissues were randomly determined.

The VMC, as a voxel element, was defined as a hexahedron of size $l_{v}$ (24.5  μm). Voxels are assigned eigenvalues linked to optical properties and addresses indicating their location, and each voxel has a 3D space with the center coordinates as the origin. Photon packets were launched orthogonally onto the center of the biological tissue model, as shown in Fig. 4, which corresponds to a collimated infinitely narrow beam of photons. The weight of the initial photon was set to 1, and the specular reflection of light was treated in the same manner as in the MCML. Because the VMC has boundaries in six directions, the condition for a photon packet to hit with the voxel boundary is as follows:

$$s-d_{b}\geq 0,$$ (5)

$$s=-\frac{\ln(\xi )}{\mu_{a}+\mu_{s}},$$ (6)

where s is the propagation distance of the photon packet in one step^25^ and $d_{b}$ is the distance to the voxel boundary. ξ is a uniformly distributed random number between 0 and 1, and $\mu_{a}$ and $\mu_{s}$ are the absorption and scattering coefficients, respectively. $d_{b}$ is calculated using the direction vector μ $\left(\mu_{x},\mu_{y},\mu_{z}\right)$ and position p $\left(p_{x},p_{y},p_{z}\right)$ of a photon packet inside the voxel as

$$d_{b}=\min\{\begin{array}{c} \frac{\frac{l_{v}}{2}-p_{x}\text{ sign}(\mu_{x})}{\left|\mu_{x}\right|}\\ \frac{\frac{l_{v}}{2}-p_{y}\text{ sign}(\mu_{y})}{\left|\mu_{y}\right|}\\ \frac{\frac{l_{v}}{2}-p_{z}\text{ sign}(\mu_{z})}{\left|\mu_{z}\right|} \end{array},$$ (7)

where a photon packet hits the voxel boundary orthogonal to the axial direction, thus yielding a minimum value for $d_{b}$ (i.e., if the expression involving $p_{x}$ and $\mu_{x}$ is the smallest compared with the other two, then the photon packet hits the boundary on the y–z plane). The positive or negative axial directions of the hitting photon packets can be determined from the direction vectors. When a photon packet hits a boundary, s is updated with

$$s_{i+1}=s_{i}-d_{b}.$$ (8)

**Fig. 5:**
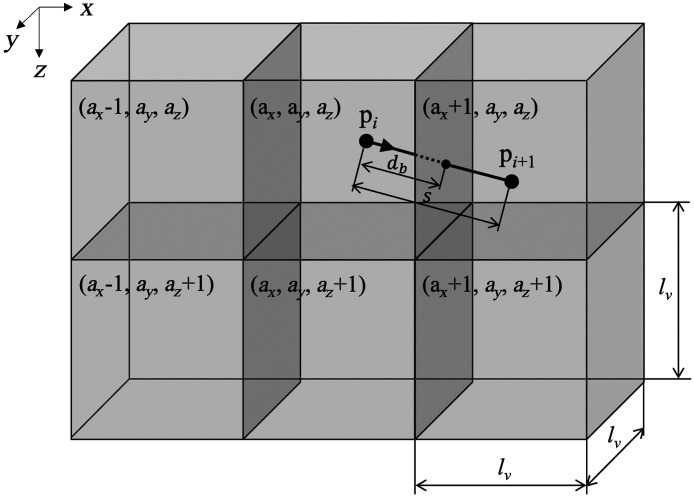
Transfer of photon packets between voxels.

If there is a difference in the refractive index between the next and current voxels, whether the photon is internally reflected or transmitted is determined by the Fresnel equations and random numbers in the same manner as in MCML. For example, when a photon packet is transmitted, the photon position is updated from $p_{i}$ to $p_{i+1}$ and address $a_{i}$ $\left(a_{x},a_{y},a_{z}\right)$ is updated to $a_{i+1}$ $\left(a_{x+1},a_{y},a_{z}\right)$ for the transmission direction, as shown in Fig. 5. Then, when the photon packet hits the boundary again, the same operations are repeated until Eq. (5) becomes negative. If a photon packet escapes from the tissue model, its position, vector, and weight are preserved.

The optical properties of the biological tissue models are listed in Table 3. The range of optical properties was determined by referring to literature values, assuming measurements with ballistic light at a wavelength of 850 nm. $\mu_{a}$ and $\mu_{s}$ of the soft tissue were determined using the measurements of Simpson et al.^36^ with uniform random numbers. The optical properties of the dermis measured by the authors included those of blacks and whites, and they assumed that the dermis and epidermis were combined. $\mu_{s}$ of the bone was derived from the equation of Ugryumova et al.^16^ Because mBMD is the wet density in Ugryumova’s equation, $\mu_{s}$ was calculated after converting mBMD to wet density from the data of Williams et al.^38^ Thus, the conversion equation between mBMD and $\mu_{s}$ is

$$\mu_{s}=17.77\mathrm{mBMD}-0.74.$$ (9)

Most of the bone marrow in the radius is composed of adipose cells after the age of 20 to 30 years.^39^ Therefore, the optical properties of the bone marrow are the same as those of the subcutaneous tissue, which is mainly composed of adipocytes.

**Table 3:** Optical properties of biological tissue models.

	$\mu_{a}$ (${\mathrm{mm}}^{-1}$)	$\mu_{s}$ (${\mathrm{mm}}^{-1}$)	g	n	Ref.
Dermis	0.0063 to 0.0856	14.20 to 25.06	0.9	1.4	Simpson et al.^36^
Subcutaneous	0.0049 to 0.0124	8.30 to 13.96	0.9	1.4	Simpson et al.^36^
Bone	0.0237	20.58	0.9	1.55	$\mu_{a}$ and $\mu_{s}$, Ugryumova et al.^16^; n, Ascenzi and Fabry^37^

$\mu_{a}$, absorption coefficient; $\mu_{s}$, scattering coefficient; g, anisotropy coefficient; n, refractive index.

### 2.3 Machine Learning

**Fig. 6:**
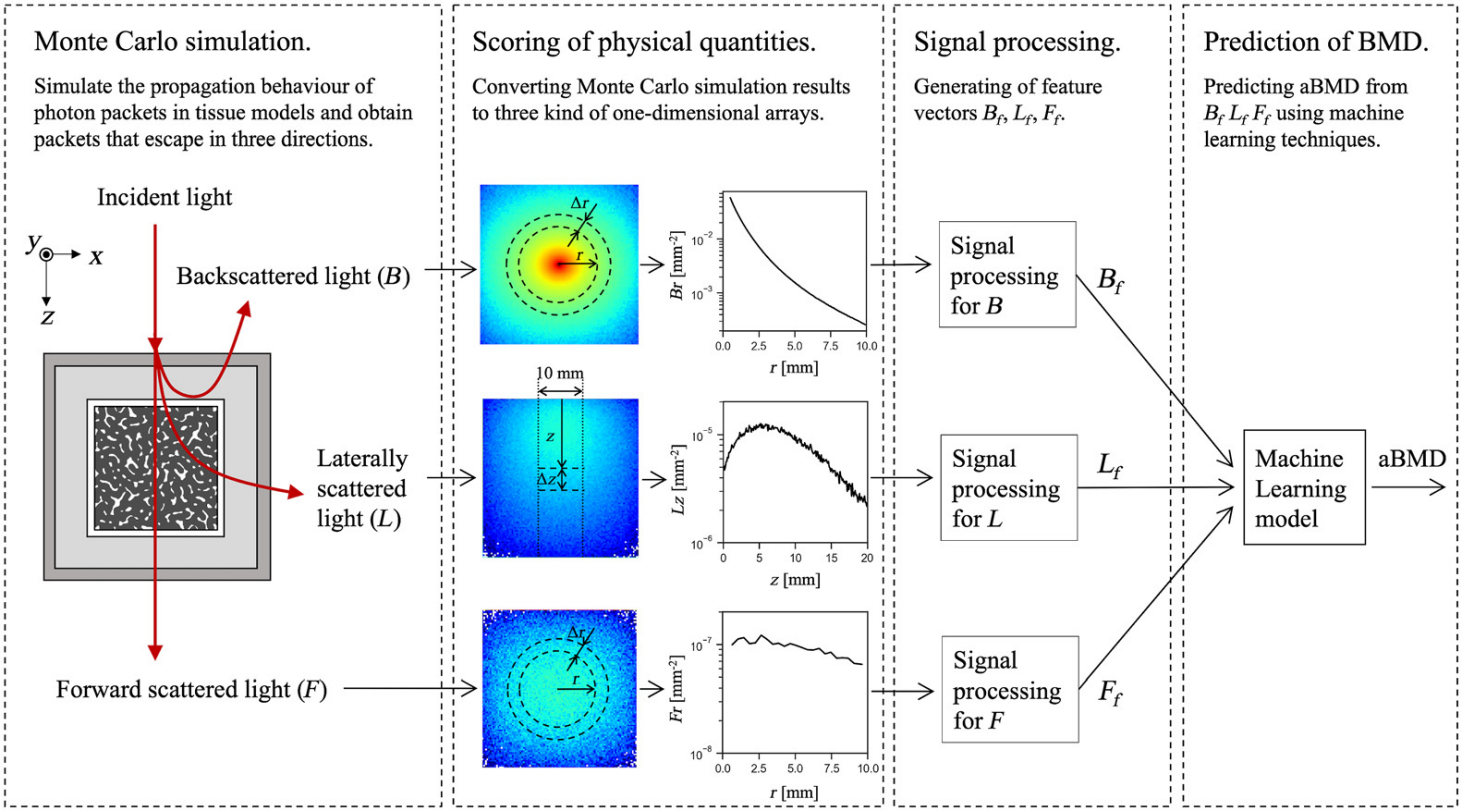
Procedure for predicting BMD using an ML model with data simulated by the Monte Carlo method.

ML techniques were used to predict the aBMD from diffuse light simulated by the VMC. An overview of the system is presented in Fig. 6. In this section, we describe the four-step calculation procedure for predicting aBMD and the module that infers the function that relates simulated diffuse light to aBMD.

#### 2.3.1 Prediction of bone density

In the first step, VMC was used to simulate light transport in a biological tissue model. Calculations were performed with a total photon count N of $10^{7}$. VMC was applied to 1211 tissue models with different structural and optical properties. In each model, the structural and optical properties were randomly combined in the ranges listed in Tables 1 and 3. Photon packets that escaped from the tissue model were categorized as backward, forward, or lateral to the direction of the packets launched. For the lateral direction, only the positive direction of the x-axis was considered. Photon packets escaping in three directions were defined as backscattered light (B), forward scattered light (F), and lateral scattered light (L).

In the second step, the physical quantity of packets escaping in the three directions was scored. For B and F, the sum of photon weights w was calculated for each radial distance r from the photon packet launched coordinate with a range of Δr, as shown in Fig. 6. Here, r is represented by the array r=[0,Δr,2Δr,3Δr,…,nΔr], and the sum of w is calculated from the range between $r_{i}$ and $r_{i+1}$. The scored light intensity arrays $B_{r}$ and $F_{r}$ were

$$B_{r}=\frac{I_{B}}{N\Delta A_{r}},$$ (10)

$$F_{r}=\frac{I_{F}}{N\Delta A_{r}},$$ (11)

where $I_{B}$ and $I_{F}$ are the arrays that represent the sum of w per $r_{i}$ with respect to B and F, respectively, and $\Delta A_{r}$ denotes the area of the annular ring, which is calculated as follows:

$$\Delta A_{r}=2\pi (i+\frac{1}{2})\Delta r^{2}.$$ (12)

$B_{r}$ and $F_{r}$ were derived up to r=10  mm with Δr=0.4  mm. For $B_{r}$, the initial position $r_{0}$ was set to 0.5 mm because the light intensity at r=0 was considered to be difficult to measure accurately when assuming actual measurements. For L, the sum of photon weights w was calculated for each positive distance along the z-axis from the photon packet irradiation coordinate with a range Δz, as shown in Fig. 6 (i.e., z is represented by the array z=[0,Δz,2Δz,3Δz,…,nΔz], and the sum of w is calculated from the range bounded by $z_{i}$ and $z_{i\;+1}$). Here, the effective width of the y-axis is ±Δy. The scored light intensity array $L_{z}$ in the z-axis direction is

$$L_{z}=\frac{I_{L}}{N\Delta A_{z}},$$ (13)

where $I_{L}$ is an array that represents the sum of w per $z_{i}$ with respect to L and $\Delta A_{z}$ indicates the area of the square, which is calculated as follows:

$$\Delta A_{z}=2\Delta y\Delta z.$$ (14)

$L_{z}$ was derived up to z=20  mm with Δz=0.1  mm and Δy=5  mm.

In the third step, the obtained $B_{r}$, $F_{r}$, and $L_{z}$ were processed to generate the feature vectors. The feature vectors $B_{f}$, $F_{f}$, and $L_{f}$ are

$$B_{f}=[B_{r},\ln\;mB_{r},\ln\;vB_{r}],$$ (15)

$$F_{f}=[\ln\;mF_{r},\ln\;vF_{r}],$$ (16)

$$L_{f}=[\ln\;L_{z},\ln\;mL_{z},\ln\;vL_{z}],$$ (17)

where $mB_{r}$, $mF_{r}$, and $mL_{r}$ are the mean and $vB_{r}$, $vF_{r}$, and $vL_{r}$ are the variances of $B_{r}$, $F_{r}$, and $L_{r}$, respectively. $F_{r}$ was not used in $F_{f}$ because $F_{r}$ did not form a valid distribution. For the $L_{z}$ signal, moving averages were calculated at 1 mm intervals. Then, to reduce the length of the $L_{z}$ array, averages of 2 mm intervals were adopted, that is, the length of array $L_{z}$ was 10. The elements of each feature vector were normalized to a mean of 0 and standard deviation (SD) of 1 for the dataset as follows:^40^

$$\frac{B_{fi}-\mu_{bi}}{\sigma_{bi}}\to B_{fi},\;i=1,2,3,\ldots ,n_{b},$$ (18)

$$\frac{F_{fi}-\mu_{fi}}{\sigma_{fi}}\to F_{fi},\;i=1,2,$$ (19)

$$\frac{L_{fi}-\mu_{l}}{\sigma_{li}}\to L_{fi},\;i=1,2,3,\ldots ,n_{l},$$ (20)

where μ and σ are the mean and SD of the dataset in each element of the feature vector and n is the total number of elements.

In the fourth step, aBMD was predicted using the ML model from $B_{f}$, $F_{f}$, and $L_{f}$. The calculations with VMC, feature vector generation, and aBMD prediction using ML models were performed in Python 3.8.

#### 2.3.2 Machine learning module

In this section, we describe the module that infers from labeled examples a function that relates the feature vectors ($B_{f}$, $F_{f}$, and $L_{f}$) and aBMD.

Four different ML techniques were tested: ridge regression (RR), support vector machine (SVM), random forest (RF), and gradient tree boosting (GTB). The criteria for selecting ML techniques were that they should be sufficiently stable and flexible for the particularities of the data, as well as have a strategy to control overgeneralization. RR solves a regression model in which the loss function is a linear least-squares function, and the regularization is given by the l2-norm.^41^^,^^42^ The coefficients of the regularization term were varied and tested in the range of $10^{-5}$ to $10^{-1}$. SVM is less sensitive to noise using ε-insensitive loss functions and can construct nonlinear functions using kernel tricks.^43^ A radial basis function (RBF) kernel was selected and tested with the kernel coefficient γ varying in the range of $10^{-4}$ to 1. The soft margin C and tube ε were also adjusted. RF is one of the decision tree-based ensemble methods in which the output is an aggregation of the outputs of a set of classification and regression trees.^44^ In RF, three parameters were adjusted: the number of decision trees, the minimum number of samples for a node to be considered a leaf, and the number of features to consider when calculating the optimal node split.^45^ GTB is one of the decision-tree-based ensemble methods and is a generalization of boosting for arbitrary differentiable loss functions.^46^^,^^47^ In GTB, six parameters were adjusted: the number of decision trees, the degeneracy of the step size used in the update to prevent overfitting, the maximum depth of the tree, the total minimum instance weights required for the children, the subsample ratio of the training instances, and the subsample ratio of the columns in building each tree.^45^ For RR, SVM, and RF, we used the modules included in scikit-learn 0.24.2, and for GTB, we used XGboost 1.4.2.

To select the best structure and parameters for each ML module, 80% of the total data were used as a training and validation dataset (trDataset). The remaining 20% of the dataset (tsDataset) were used for testing and comparison purposes. The structure and parameter set of each algorithm were modified, and the best performing one was selected after a 10-fold cross-validation with grid search in trDataset. In 10-fold cross-validation, 90% of the dataset was randomly selected and used for training, and the remainder was used for validation. This was performed 10 times by rotating the dataset. The stability of the ML techniques was also verified by cross-validation. After obtaining the best configuration, tsDataset was used to evaluate the performance. The coefficient of determination ($r^{2}$) was used as the metric for performance evaluation.

## 3 Results

**Fig. 7:**
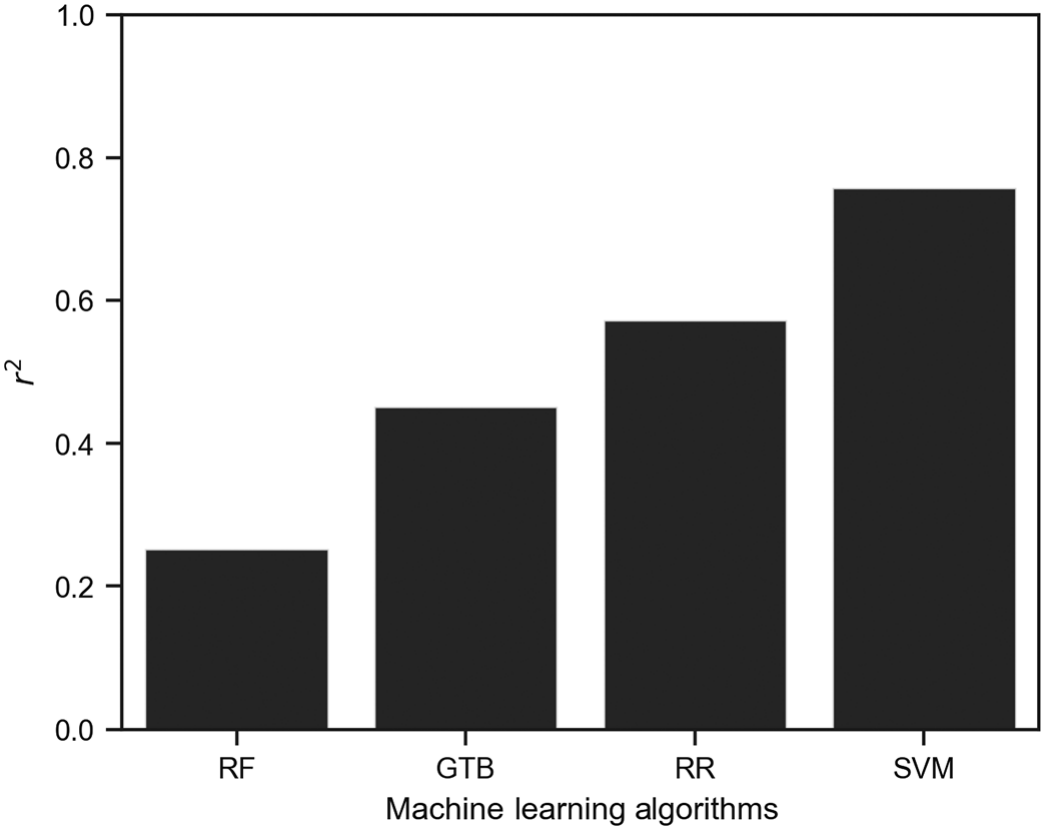
Comparison of aBMD prediction performance by different ML algorithms. SVM, support vector machine; RR, ridge regressor; GTB, gradient tree boosting; RF, random forest.

The criterion for selecting the ML algorithm was the value of the coefficient of determination $r^{2}$ on tsDataset. tsDataset is a random selection of 20% (242) of the original dataset. The trDataset, which contains the remaining 80% (969), was used to train the algorithms. The ML algorithm was tuned in trDataset to achieve the best performance with 10-fold cross-validation. Once the best set of parameters and structures were found, the ML algorithms were retrained with the trDataset, and the performance was tested using tsDataset. Figure 7 shows a comparison of the coefficients of determination for each algorithm. The SVM regression exhibited the best performance ($r^{2}=0.757$). The coefficients of determination for the other ML algorithms were $r^{2}=0.572$ for RR, $r^{2}=0.451$ for GTB, and $r^{2}=0.252$ for RF. To determine the stability of the algorithm for unknown data, the SD of $r^{2}$ between the 10-fold cross-validation at trDataset was computed. The SD of $r^{2}$ in the cross-validation of SVM was 0.056, which is an acceptable value.

**Fig. 8:**
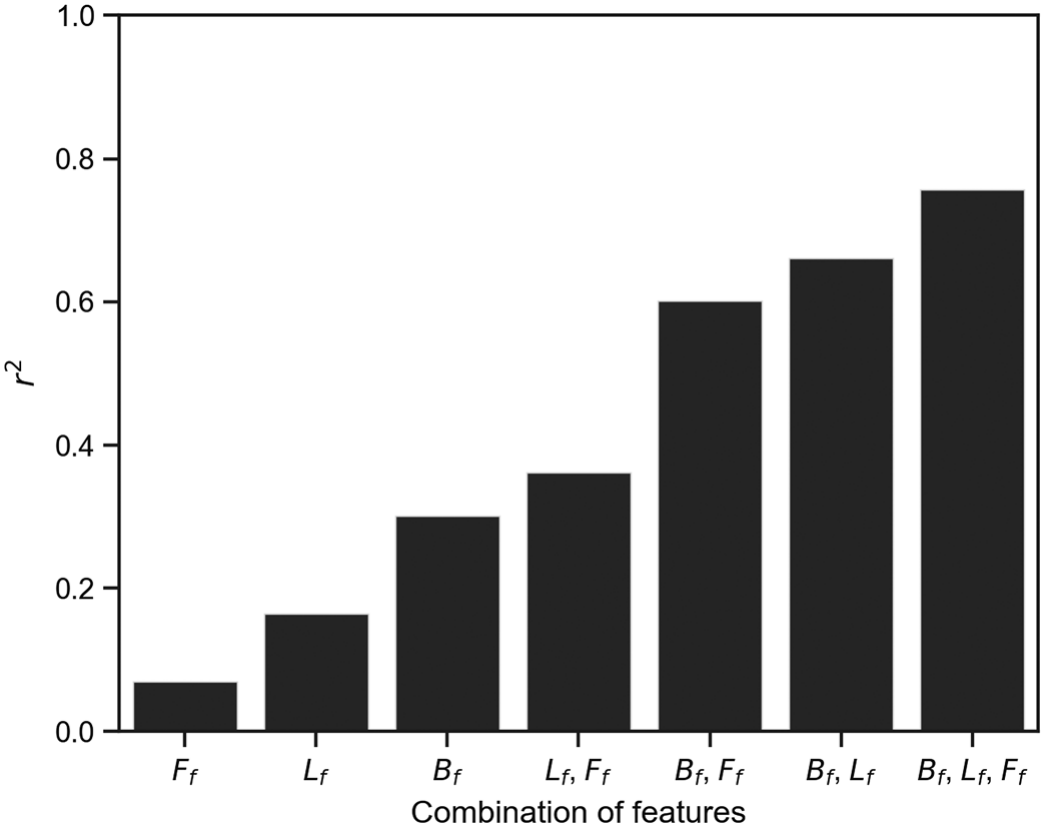
Differences in the coefficient of determination ($r^{2}$) of SVM regression by combinations of feature vectors. $B_{f}$, feature vector from backscattered light; $L_{f}$, feature vector from lateral scattered light; $F_{f}$, feature vector from forward scattered light.

For the prediction of aBMD using SVM, the coefficient of determination $r^{2}$ on tsDataset was computed for all combinations of $B_{f}$, $L_{f}$, and $F_{f}$ (Fig. 8). The purpose of this study was to investigate the extent to which feature vectors and their combinations are related to aBMD. The parameters were tuned for all feature vector combinations with 10-fold cross-validation in trDataset. The prediction combining all of the feature vectors showed a coefficient of determination. Conversely, the prediction of aBMD from $B_{f}$, $L_{f}$, and $F_{f}$ alone exhibited lower performance ($r^{2}\leq 0.302$). These results suggest that it is difficult to predict aBMD with one feature vector, but combining feature vectors allows for highly accurate predictions.

**Fig. 9:**
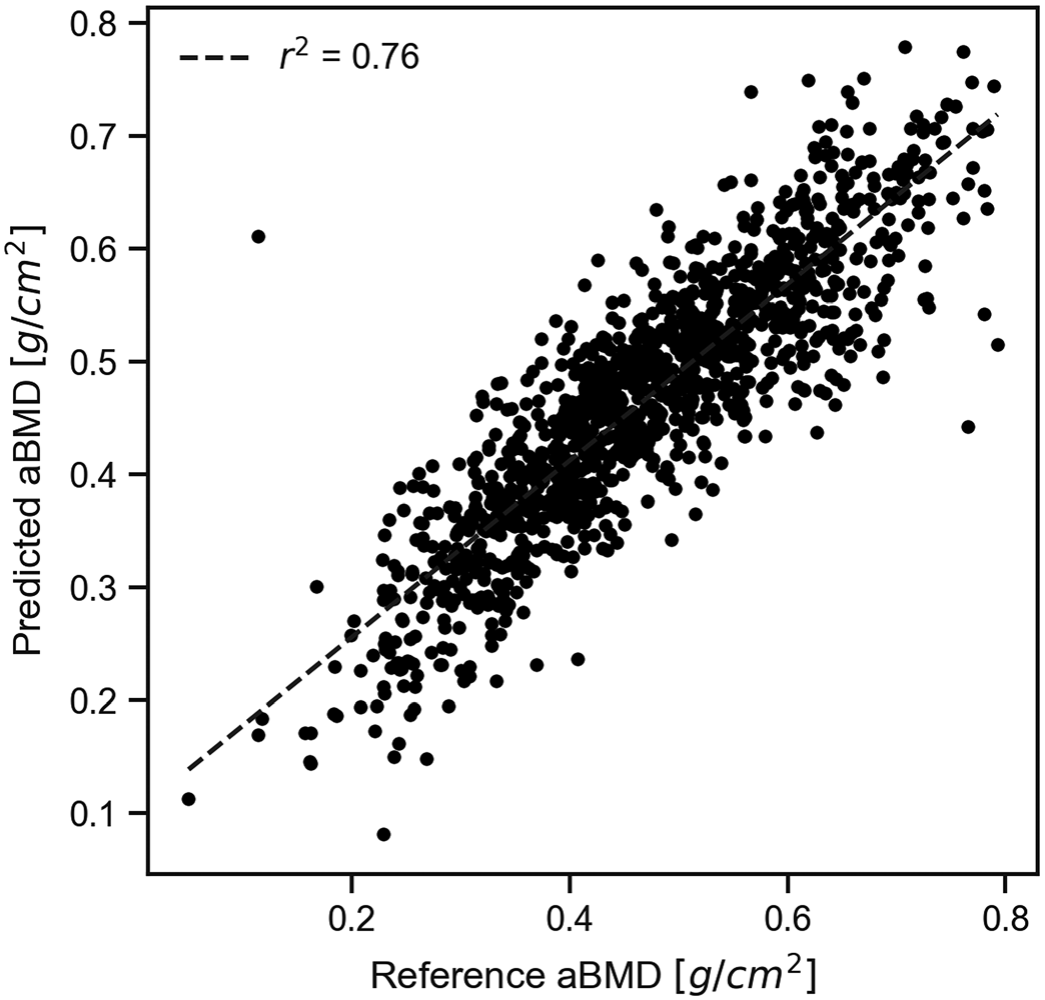
Relationship between predicted and reference aBMD.

**Fig. 10:**
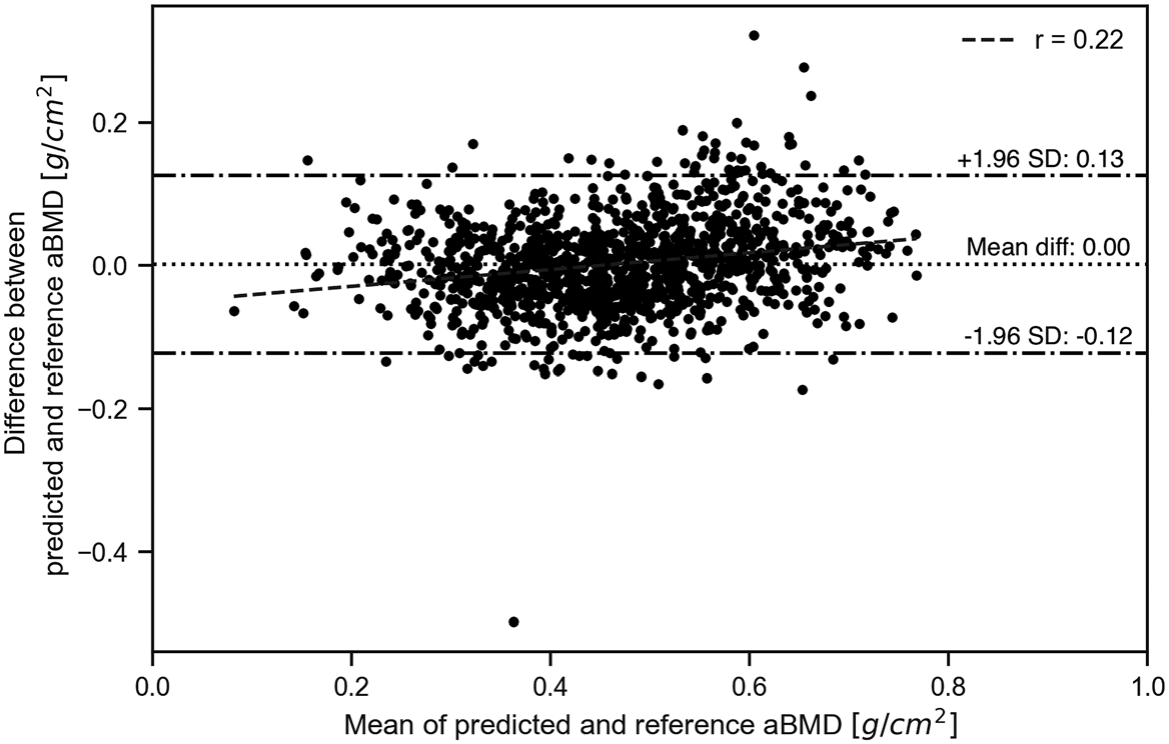
Bland–Altman plot for predicted and reference aBMD.

As the final test, the performance of the system was tested on all 1211 data cases using the SVM method. SVM was selected because it gave the highest $r^{2}$ value for the aBMD prediction. The performance was assessed using 10-fold cross-validation on the entire dataset. The relationship between the predicted and reference values of aBMD is shown in Fig. 9. A linear regression of predicted and reference aBMD yielded an $r^{2}$ value of 0.760, indicating reasonable agreement. The Bland–Altman (BA) plot is shown in Fig. 10. The BA plot is a method used to check the agreement and systematic errors between two measurement methods.^48^ The BA plot indicates a moderate correlation coefficient r of 0.22, with a slight proportional bias. This proportional bias may not be a problem in practice. The mean difference between the predicted and reference values was 0.00, with no fixed bias. The limit of agreement for the predicted values was $\pm 0.124\;g/{\mathrm{cm}}^{2}$. These results suggest that BMD can be predicted with high accuracy using this method, even if there is variance in the thickness and optical properties of the soft tissue.

## 4 Discussion

The purpose of this study was to develop an optical bone densitometry method that is insensitive to individual differences in the structural and optical properties of soft tissues and to verify its feasibility. In the proposed method, spatially resolved diffuse light was obtained in three directions (backward, forward, and lateral to the direction of light irradiation) simulated by the Monte Carlo method, and BMD was predicted using ML techniques from these data. The cross-validation demonstrated that the proposed method can predict BMD with high accuracy and low error in the simulation. The results suggest that the proposed method can be applied to optical bone densitometry, which is robust to variations in soft tissue.

Because the biological tissue model synthesized in this study is randomly constructed based on the range that a living organism can exhibit, the data obtained by the Monte Carlo simulation are considered to reflect a population with sufficient variance. The range of soft tissue properties was determined randomly based on measurements. In particular, the optical properties of the dermis cover a wide range of people, from colored individuals to Caucasians.^36^ In bone tissue, a trabecular pattern was generated by the reaction-diffusion model because it is difficult to assign the trabecular bone a single scattering coefficient representative of a BMD value. The equation of Ugryumova et al.^16^ yields negative scattering coefficients for a range of trabecular bone BMDs. In addition, Pifferi et al. found no age-related changes in the scattering coefficient in calcaneal measurements using TSR.^19^ Overall, the unique and irregular shape of the trabecular pattern may lead to a different light scattering process compared with a homogeneous medium. The trabecular patterns generated in this study were similar to those of real bone in terms of appearance, BV/TV, and quantitative geometric structure. In addition, BMD was randomly adjusted from severe osteoporosis to the range of normal subjects. Moreover, the Monte Carlo method is the gold standard for modeling light transport in tissues.^49^ Therefore, we consider that the diffuse light simulated in this study represents the structural and optical properties of the biological tissue for a population of sufficient variance.

The results shown in Fig. 7 seemingly contradict those reported by Chung et al.^14^ Those authors demonstrated that the transmitted light intensity and aBMD are strongly correlated in the measurement of the ultradistal radius using an 850 nm wavelength light. However, in our results, the aBMD estimated only from transmitted light does not show a high coefficient of determination. This inconsistency seems to be due to the dispersion of the population. Chung et al. focused on a limited sample size of (10 participants). BMD measurements using only transmitted light are probably limited to populations with small variations in soft tissue composition. Nevertheless, $F_{f}$, when combined with R, $R_{f}$, and $L_{f}$, clearly increased the coefficient of determination. This result suggests that the combination of light measured in different directions reduces the error from soft tissue with individual differences.

There is a possible nonlinear relationship between BMD and spatially resolved diffuse light observed in several directions. As shown in Fig. 8, SVM showed a higher prediction performance than RR. The RR is an algorithm based on linear multiple regression,^41^^,^^42^ whereas SVM is an algorithm that can be applied to nonlinear data by mapping using nonlinear kernel functions, such as RBF.^43^ In other words, the difference in the coefficient of determination between RR and SVM predictions of aBMD is probably due to the difference in the ability to support nonlinearity. GTB and RF are also nonlinear algorithms, but because they are decision-tree-based algorithms, they are probably not suitable for application to diffuse light, which shows continuous variation with distance from the light source. Therefore, when predicting BMD from our data, an algorithm for nonlinear data, such as SVM, is considered necessary.

The aBMD predicted using SVM from the Monte Carlo simulated data had a high coefficient of determination and lower error (Figs. 9 and 10). Thus, it has been demonstrated that our method can evaluate BMD with high prediction performance even when the bone is surrounded by soft tissue with individual differences in thickness and optical properties.

We acknowledge that there are several limitations to this study. First, the simulation was only a theoretical test. However, the simulations provide theoretical and clear insights into the different tissues that affect diffuse light. In addition, we believe that the combination of Monte Carlo simulation and ML techniques has several implications for the development of noninvasive medical measurements using the light diffusion theory that are beyond theoretical verification. An ML model built with sufficient variance and a large amount of data has excellent generalization performance; however, in the field of medical measurement, there are often ethical barriers and difficulties in data acquisition, such as a limited number of cases and invasive measurements. The simulation, which can generate almost unlimited data if computational resources are available, could offer a promising solution to such problems. Second, the VMC has boundaries in only six directions, which is an obvious limitation when compared with mesh-based methods^49^^–^^52^ that can represent more free boundaries. However, inside a light-scattering medium, such as a biological tissue with a complex structure, light is averaged by scattering. Therefore, an overly accurate representation of the curvature of the trabecular structure is probably not practical. Third, a simple rectangular biological tissue model was adopted in this study. This model did not consider the heterogeneity in soft tissues, blood vessels, and complex bone structures; however, this information could be implemented in the model using CT and MRI techniques. However, the potential importance of this model is that it allows for a simple and targeted discussion of random changes in soft tissue thickness and optical properties in the validation of optical bone densitometry. This model might provide useful information about the interaction between the bone and soft tissue in measurements using diffuse light. All of the limitations mentioned here are attributed to the fact that it is unclear how much of the actual biological tissue should be assumed in the model to represent the light diffusion phenomenon and its output *in vivo*. It is also possible that simpler models represent substantial physical phenomena. Therefore, it is necessary to verify this by model experiments using phantoms and clinical trials.

## 5 Conclusion

In this study, we developed optical bone densitometry using Monte Carlo simulation and ML techniques and validated this method by cross-validation. From the results obtained, we concluded that the aBMD predicted from spatially resolved steady-state diffuse light data acquired in the backward, forward, and lateral planes of the biological tissue model was robust to differences in soft tissue layers thickness and optical properties.

## Code, Data, and Materials Availability

The codes, data, and materials that support the findings of this study are available from the corresponding author upon reasonable request.
